# Retraction Note: Cardiac arrest triggers hippocampal neuronal death through autophagic and apoptotic pathways

**DOI:** 10.1038/s41598-025-91288-2

**Published:** 2025-02-25

**Authors:** Derong Cui, Hanbing Shang, Xiaoli Zhang, Wei Jiang, Xiaofeng Jia

**Affiliations:** 1 Department of Anesthesiology, Shanghai Sixth People’s Hospital Affiliated With Shanghai Jiaotong University, Shanghai, 200233 China; 2 Department of Anesthesiology and Critical Care Medicine, Johns Hopkins University School of Medicine, Baltimore, MD 21287 USA; 3 Department of Neurosurgery, Shanghai Ruijin Hospital Affiliated With Medical School of Shanghai Jiaotong University, Shanghai, 200025 China; 4 Biomedical Engineering, Johns Hopkins University School of Medicine, Baltimore, MD 21205 USA; 5 Department of Neurosurgery, University of Maryland School of Medicine, Baltimore, MD 21201 USA; 6 Department of Orthopaedics, University of Maryland School of Medicine, Baltimore, MD 21201 USA

Retraction of: *Scientific Reports* 10.1038/srep27642, published online 08 June 2016

The Editors have retracted this Article. Following the publication of this article, image concerns were raised regarding similarities between panels within this Article and with a previously published article [1]

- Figure1B and 1E LC3-I and LC3-II blots.
- Figure 2I, 5B, and 5E GAPDH blots.
- Figure 4B GAPDH (from lanes 3–6) and 6E GAPDH (from lanes 1–4) blots.
- Figure 7A and 7C hippocampus sections in CA+BFA, CA+3-MA (3h after ROSC) and CA+3-MA (6h after ROSC) groups.
- Figure S4 CA+DMSO and CA+3-MA (1.5 h after ROSC) all subpanels.
- Multiple overlaps between Figures 7A & 7C and Figures 8A, C, E, F, and G of [1].
- Multiple overlaps between Figures S1A & S1B and Figures 10A and 10B of [1].

The Authors were able to provide some of the original data upon request but that did not resolve all the concerns. The Editors therefore no longer have confidence in the data underlying this Article.

Xiaofeng Jia agrees with this retraction. Derong Cui has not explicitly stated if they agree or disagree with this retraction. None of the remaining Authors has responded to any correspondence from the Editor or Publisher about this retraction.
